# Common Beans as a Source of Amino Acids and Cofactors for Collagen Biosynthesis

**DOI:** 10.3390/nu15214561

**Published:** 2023-10-27

**Authors:** Carolina Añazco, Paola G. Ojeda, Marion Guerrero-Wyss

**Affiliations:** 1 Laboratorio de Bioquímica Nutricional, Escuela de Nutrición y Dietética, Carrera de Nutrición y Dietética, Facultad de Ciencias para el Cuidado de la Salud, Universidad San Sebastián, General Lagos #1190, Valdivia 5110773, Chile; 2 Instituto de Ciencias Aplicadas, Universidad Autónoma de Chile, Talca 3460000, Chile; paola.ojeda@uautonoma.cl

**Keywords:** amino acids, cofactor, collagen, common beans, lysine, *Phaseolus vulgaris* L., vegan diets

## Abstract

Common beans (*Phaseolus vulgaris* L.) are widely consumed in diets all over the world and have a significant impact on human health. Proteins, vitamins, minerals, phytochemicals, and other micro- and macronutrients are abundant in these legumes. On the other hand, collagens, the most important constituent of extracellular matrices, account for approximately 25–30 percent of the overall total protein composition within the human body. Hence, the presence of amino acids and other dietary components, including glycine, proline, and lysine, which are constituents of the primary structure of the protein, is required for collagen formation. In this particular context, protein quality is associated with the availability of macronutrients such as the essential amino acid lysine, which can be acquired from meals containing beans. Lysine plays a critical role in the process of post-translational modifications facilitated with enzymes lysyl hydroxylase and lysyl oxidase, which are directly involved in the synthesis and maturation of collagens. Furthermore, collagen biogenesis is influenced by the cellular redox state, which includes important minerals and bioactive chemicals such as iron, copper, and certain quinone cofactors. This study provides a novel perspective on the significant macro- and micronutrients present in *Phaseolus vulgaris* L., as well as explores the potential application of amino acids and cofactors derived from this legume in the production of collagens and bioavailability. The utilization of macro- and micronutrients obtained from *Phaseolus vulgaris* L. as a protein source, minerals, and natural bioactive compounds could optimize the capacity to promote the development and durability of collagen macromolecules within the human body.

## 1. Introduction

The common bean, *Phaseolus vulgaris*, is often consumed because of its substantial nutritional value [1]. This legume provides a significant source of dietary fiber, unsaturated fatty acids, vitamins, minerals, carbohydrates, phenolic compounds, and proteins [2]. A balanced diet that contains beans is essential in reducing the complications of noncommunicable chronic diseases including diabetes and cardiovascular diseases, which are on the rise [3,4,5,6].

Common beans contain a greater quantity of essential amino acids, including lysine, the most abundant amino acid in dry beans, as well as tyrosine and phenylalanine [1,4,7]. In particular, the protein composition of different bean cultivars ranges from 15% to 35% [8,9,10]. Bean extracts that are abundant in phenolic compounds have demonstrated various beneficial properties such as antioxidant, antiglycating, antimutagenic, anti-inflammatory, chemopreventive, antibacterial, antiaging, and anti-diabetic activities [11,12]. It is commonly accepted that the presence of bioactive chemicals contributes to the beneficial effect on human health [13,14].

**Figure 1:**
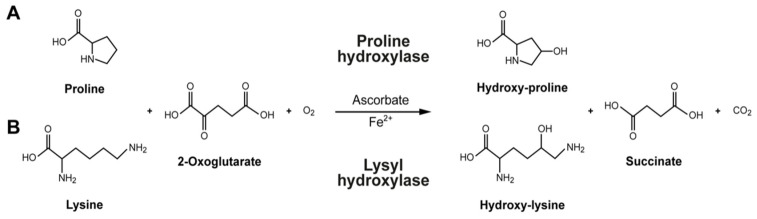
**Formation of collagen amino acids:** (**A**) The intracellular proline modification to hydroxyproline using proline hydroxylase. (**B**) The intracellular lysine modification to hydroxylysine using lysyl hydroxylase. Ascorbate (vitamin C) and iron act as cofactors for the proline and lysine hydroxylases that stabilize the tertiary structure of collagen molecules.

Additionally, beans are among the legumes with the highest vitamin and mineral concentrations, making them a superior source of micronutrients [1,15]. The common bean does include some water-soluble cofactors, such as ascorbate (vitamin C) and minerals like copper (Cu) and iron (Fe) [16]. Vitamin C and iron are required for the conversion of lysine to hydroxylysine and proline to hydroxyproline, which are subsequently incorporated into collagen (Figure 1) [17]. For instance, iron and vitamin C deficiencies can alter the enzymatic function of hydroxylase enzymes, and this dysregulation can result in collagen defects associated with scurvy [18,19,20]. Because they function as cofactors for enzymatic activities necessary to generate fiber scaffolds to create the extracellular matrix in mammalian tissues, these micronutrients are essential for collagen synthesis [21].

**Figure 2:**
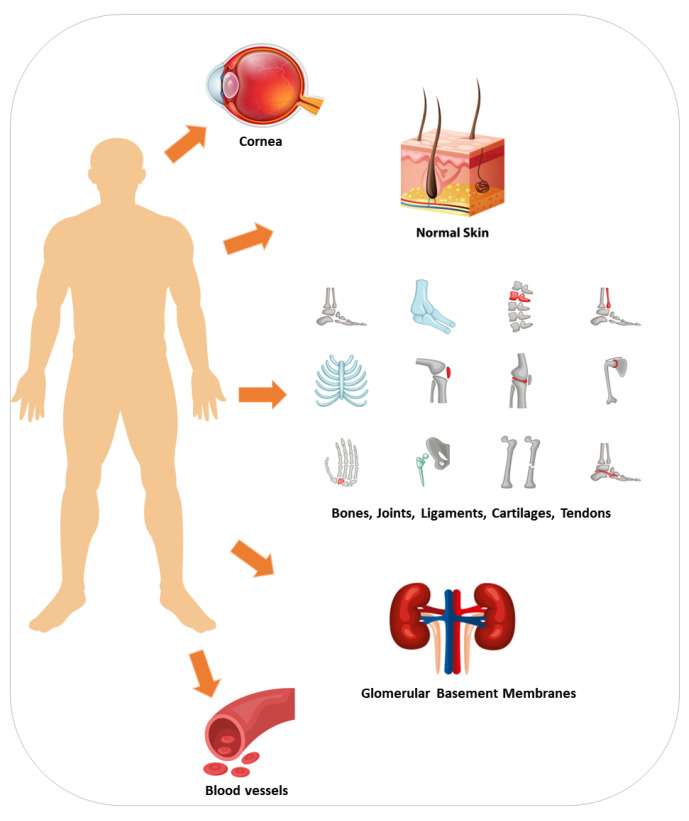
**Distribution of collagen in various tissues:** Collagen is a component of the extracellular matrix, and it gives tissues such as skin, bones, tendons, ligaments, cartilage, cornea, and blood vessels the strength, support, and integrity they need to function properly.

Collagen is the most prevalent and essential structural protein expressed in the human body, serving as the fundamental scaffold of connective tissues such as skin, cartilage, and bone (Figure 2) [22,23]. It plays a crucial role in establishing an appropriate tissue architecture as a component of the extracellular matrix that surrounds cells [18,23]. Critical aspects for controlling tissue genesis and homeostasis are provided with the structure of extracellular tissues [24,25]. Collagen production naturally decreases with age, leading to changes in skin elasticity, joint health, and other connective tissues. Due to its various benefits, collagen has become a common supplement in the pharmaceutical industry to support skin protection, joint function, and overall health [26].

According to reports from Grand View Research, the global collagen market size was valued at USD 9.12 billion in 2022. Collagen is sourced from various animal tissues, such as cow or fish skin, and is also produced synthetically for medical and cosmetic purposes due to the increased demand for plant-based collagen [27]. In order to respond to the requirements of a growing market segment characterized by a preference for vegetable-based alternatives, such as consumers adhering to vegetarian and vegan diets, it becomes imperative to engage in the development of innovative substances that may effectively substitute animal-derived components, such as gelatin.

In light of these findings, this review considers the published research on the potential effects of macro- and micronutrients derived from *Phaseolus vulgaris* L. on collagen biogenesis. We also outline how amino acids, vitamins, and minerals found in ordinary beans can help the body produce collagen. Also, the purpose of this article is to evaluate the current understanding of the relationship between collagen function and selected micro- and macronutrients contained in *Phaseolus vulgaris* L. as collagen options for vegetarians. There are no vegetarian sources of collagen protein. Common bean peptides offer an opportunity for establishment of animal-free substitutes to gelatin in the food and beverage sectors, particularly in the context of vegan dietary practices.

## 2. Health Benefits Associated with the Consumption of Common Beans

The recommendation for the consumption of legumes, including beans, defined by the World Health Organization (WHO) corresponds to a minimum consumption of twice a week, while in some countries, even official organizations suggest increasing consumption to 3–4 weekly servings, considering a serving as 60–80 g of raw legumes, to ensure benefits for human health [28]. These recommendations are equivalent to a minimum legume consumption of 25.7–45.7 g/day.

It has been demonstrated that health benefits associated with the consumption of legumes are diverse, due to their nutritional profile, consideration as a food high in dietary fiber, slowly absorbed carbohydrates, and a low contribution of total lipids (Table 1) [29]. The polyphenol content present in legumes, especially in *Phaseoulus vulgaris*, has been associated with anticancer activity, considering the consumption of this food as part of the usual diet [30,31,32]. *Phaseolus vulgaris* contains a wide variety of phytochemicals with antioxidant activity, such as phenolic acids, tannins, flavonoids, flavanols, isoflavones, anthocyanins, and proanthocyanidins [9,33,34]. Various authors have described that the consumption of beans is also associated with antioxidant potential, and antimutagenic and antiproliferative activities [31,35]. Furthermore, the profile of bioactive compounds, particularly total phenolics, individual phenolic acids, flavonoids, anthocyanins, and tannins, in addition to a limited contribution of fatty acids (but of very good quality; monounsaturated and polyunsaturated fatty acids) give a unique value to the bean as a food for regular consumption with a protective effect in metabolic syndrome, at the endothelial level, reducing endothelial damage in the development of atherosclerosis and inflammation, and preventing complications such as acute myocardial infarction, which corresponds to one of the leading causes of mortality in adults worldwide [36].

A study with atherogenic mice supplemented with a fresh-ground bean protein hydrolysate, equivalent to approximately one daily serving of cooked beans, showed significant decreases in plasma triglycerides and total cholesterol after 9 weeks [37]. In addition, improvements in inflammation and endothelial dysfunction demonstrated 62% increased endothelial nitric oxide synthase (e-NOS) and a 57% nitric oxide serum concentration, in addition to gene expression changes in TNFα (94% reduction) and angiotensin II (79% reduction), as compared to an atherogenic diet alone [37].

In addition, bean consumption has been associated with a significant reduction in cardiovascular risk and mortality from cardiovascular events, due mainly to its reducing effect on cholesterol and glycemia levels and the risk of type 2 diabetes [38,39,40].

At the same time, bean consumption has been shown to be associated with lower rates of coronary heart disease through weight reduction and prevention of obesity in subjects who consume beans as part of their usual diet. In this sense, overweight individuals who received a *Phaseolus vulgaris* L. extract had a significantly greater reduction in the body weight index, fat mass, adipose tissue thickness, and anthropometric measurements of the waist, hip, and thigh compared to the placebo group [29,41,42]. The methanolic extract of *Phaseolus vulgaris* L. has been considered relevant by its antiplatelet effect, particularly the ability to suppress platelet secretion, using the proposed mechanism of protein kinase A (PKA) modulation and the inhibition of AKT phosphorylation [43].

It is interesting to discuss the *Phaseolus vulgaris* L. agglutinin production of nitric oxide (NO), regulated by the Ca^2+^/calmodulin kinase/AMPK pathway in a time- and dose-dependent way. This process is dependent on the eNOS phosphorylation involving the eNOS/NO/cGMP/PKG pathway [44,45]; the NO production by the beans’ agglutinin can decrease the platelet aggregation, explaining the lower platelet activation in comparison to agglutinin from whole grain [46].

The common bean hydrolysate has revealed numerous effects from an angiotensin-converting enzyme inhibitor to an antimicrobial, antioxidant, and even tumor cell inhibitor. The bioactive potential of peptides presents in the indigestible fraction of common beans that protect cells from oxidative stress and inhibit the angiotensin-I-converting enzyme by interacting with its catalytic cavity independently of its antioxidant capacity was documented [47]. Gomes et al. explained the hydrolysate capacity to modulate lipid metabolism and prevent endothelial dysfunction in BALB/c mice; they also showed hypocholesterolemic activity, helping to reduce inflammation, oxidative stress, and endothelial dysfunction [37]. In addition, the use of hydrolysates of *Phaseolus vulgaris* shows a promising effect in mice, from the modulation of the lipid profile to the increase in e-NOS expression [37]; this effect can be explained with the effect of the compounds found in the bean, upon endothelial cells. As is the case of amino acids such as lysine, leucine, serine, and glutamine that work as modulators of NO production [48].

The common bean consumption includes multifactorial gastrointestinal physiological mechanisms such as facilitation of nutrient transit through the digestive tract, butyric acid production in the colon, and absorption and/or dilution of substances in the gut [49]. On the other hand, prebiotic compounds, including dietary fiber found in beans, benefit human health by providing protection against the development of certain diseases, potentially via modulation in gut microbial composition [50,51,52,53].

Quercetin, a vegetal flavonoid, present in beans has been shown to have anti-inflammatory properties and does so by inhibiting the cyclooxygenase pathway [54]. Even quercetin has also been shown to inhibit the growth of Helicobacter pylori bacteria in in vitro studies [54,55,56]. Methyl-3-(+)-catechin interferes with the formation of histamine in gastric mucosa and hence produces a protective effect. Most flavonoids have anti-viral effects against Herpes simplex virus, respiratory syncytial virus, parainfluenza virus, and adenovirus.

*Phaseolus vulgaris* L. is a legume with hypoglycemic and antioxidant effects and prevents kidney damage in a diabetes model. However, until now, the molecular mechanism of this protection has not been elucidated. To evaluate the effect of bean consumption, diabetic animals were fed for 4 weeks with food supplemented with 10% beans. Some bean varieties decreased glucose levels in diabetic rats, as well as some markers of kidney damage in the serum and urine. Regarding the expression of genes related to kidney function, bean consumption increased the expression of some genes, such as threonine protein kinase 1 (pim-1), and arginine succinate lyase (asl), and decreased the expression of carbamoyl phosphate synthase subunit 1 (cps1) and inositol polyphosphate multikinase (ipmk), among others. These genes could be related to the elimination of amino groups, regulation of creatinine production, and decreased risk of metabolic acidosis. These results suggest that the consumption of cooked common beans can be used as an alternative to regulate kidney damage associated with diabetes [57]. In another study in diabetic rats, bean consumption produced a hypoglycemic and hypolipidemic effect in early-stage diabetic rats, while in diabetic animals of the advanced stage, a decrease in glucose levels was not observed, but a decrease in markers of kidney damage such as protein and albumin in urine was, in addition to total cholesterol, LDL cholesterol, IL-6, and TNF-α [58].

Finally, the influence that is exerted with an extract of *Phaseolus vulgaris* on collagen content in streptozotocin-diabetic rats has been reported, showing a significant decrease in the deposition of collagen in diabetic rats [5]. However, additional studies are necessary to clarify the effect of *Phaseolus vulgaris* extracts on collagen content or amino acid modifications in healthy models.

**Table 1:** The most important benefits for health from *Phaseolus vulgaris*.

Nutrients/System	Beneficial Effect	References
Beans’ nutritional profile as healthy food has high content of dietary fiber and slowly absorbed carbohydrates	Reduces glucose plasma levels and decreases the type 2 diabetes risk	[29,40]
Phytochemical content: Phenolic acids, tannins, flavonoids, flavanols, isoflavones, anthocyanins, and proanthocyanidins	Antioxidant activity, and antimutagenic and antiproliferative activities	[9,31,33,34,35]
Total phenolics, individual phenolic acids, flavonoids, anthocyanins, and tannins, in addition to a limited contribution of fatty acids	Protective effect in metabolic syndrome In endothelium, reduces damage in the development of atherosclerosis and inflammation	[13,36,37]
Fatty acid metabolism	Improves the lipid profile: Reduces total cholesterol and LDL and increases HDL cholesterol.	[36,38]
Weight and obesity	Reduction in body weight index, fat mass, adipose tissue thickness, and anthropometric measurements of waist, hip, and thigh	[29,41,42]
Vascular system	Antiplatelet effect, and the ability to suppress platelet secretion	[43,46]
Peptides present in the indigestible fraction of common beans	Protect cells from oxidative stress and inhibit the angiotensin-I-converting enzyme	[47]
Hydrolysates and amino acids (lysine, leucine, serine, and glutamine)	Increase the e-NOS expression and module of NO production	[37,48]
Dietary fiber and gut system	Facilitation of nutrient transit, probiotic effect, modulation in gut microbial composition	[49,50,51,52,53]
Quercetin	Anti-inflammatory properties	[54]

## 3. What Is the Protein Collagen?

The protein collagen is widely distributed throughout the human body and plays an important function in the structural maintenance of many different types of tissue, including skin, bones, and connective tissue [22]. The term “collagen” has its etymological roots in the Greek language, specifically derived from the combination of two words: “kola,” which translates to gum, and “gen,” which signifies generation [59]. The amalgamation of these two lexical units results in the English expression “collagen.” The extracellular matrix is comprised of this substance, which contributes to the structural integrity and support of various tissues, including but not limited to skin, bones, tendons, ligaments, cartilage, and blood vessels [18]. The collagen protein consists of amino acids, with glycine, proline, and hydroxyproline being the primary constituents [60]. There are different types of collagens found in the body; so far, there are 28 types of collagens reported that contain at least one triple-helical domain [59]. For example, type I collagen is found in skin, bones, tendons, ligaments, and organs (Figure 2). It provides tensile strength and is the most abundant type of collagen in the body; type II collagen is primarily found in cartilage, providing support and flexibility; type III is found together with type I collagen in skin, blood vessels, and organs [61]. It helps support the structure of organs and tissues, and type IV is found in the basement membrane, providing support for cell adhesion and filtration [23,24]. The core of the collagenous domain contains Xaa-Yaa-Gly triplets, where glycine is present at every third position in the sequence. This arrangement of amino acids contributes to the strength and stability of collagen [62]. Typically, the Xaa and Yaa positions are occupied by proline and hydroxyproline, respectively. These amino acids play a crucial role in facilitating the establishment of interchain hydrogen bonds, thereby enhancing the stability of the triple helix structure [60,63]. Additionally, several other amino acids, such as lysine, arginine, glutamate, and aspartate, are involved in electrostatic interactions with type I procollagen [22].

**Figure 3:**
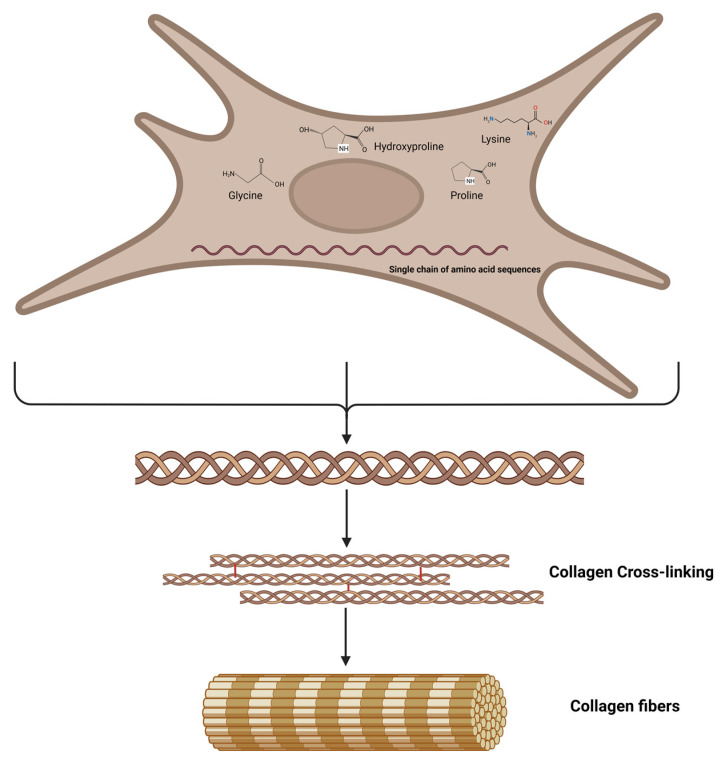
**Schematic representation of the collagen biosynthesis:** Procollagen molecules convert to trimeric propeptide fragments that form tropocollagen molecules. Tropocollagen molecules self-assemble via the reaction of aldehyde groups and the formation of covalent bonds that cross-link collagen molecules into fibrils and fibers.

Lysine is known to have a significant impact on the post-translational modifications facilitated with enzymes, namely lysyl hydroxylase and lysyl oxidase. These enzymes are directly implicated in the synthesis and maturation of collagen [64,65,66,67,68,69,70]. Enzymes such as lysyl hydroxylase and lysyl oxidase play a crucial role in various biological processes, including the biosynthesis, secretion, and maturation of collagens. These enzymes achieve this by post-translationally modifying lysine residues within collagen structures, which can be found in both network and fiber forms [69]. The process of lysine modification within the cell is facilitated with the enzyme lysyl hydroxylase, which catalyzes the hydroxylation of specific peptidyl lysine residues, resulting in the production of hydroxylysine. Furthermore, it has been observed that the hydroxylysine residues located in the helical domain have the potential to undergo glycosylation through the addition of galactose and glucose residues [69,71]. Hydroxylysine is exclusively present in proteins derived from animals and humans, primarily within collagen macromolecules. However, it is also present in the collagen-like region of various proteins distinct from collagens [61]. Lysyl oxidases are responsible for catalyzing enzymatic oxidative deamination on the ε-group of lysine and hydroxylysine residues, resulting in the production of reactive aldehydic residues or allysine [72]. Subsequently, reactive aldehydes initiate a sequence of non-enzymatic condensation reactions, leading to the formation of distinct covalent intra- and intermolecular cross-links between triple-helical chains. These cross-links play a vital role in stabilizing mature collagen, thereby contributing significantly to the biomechanical properties of the collagen fiber (Figure 3) [73].

## 4. Amino Acids to Support Collagen Production

Nutritionally non-essential amino acids such as glycine, proline, and hydroxyproline can be generated with endogenous synthesis in mammals, and their contribution to the total amino acid content in collagen is around 57% [60]. Also, it has been demonstrated that proline and its precursor, glutamate, significantly enhance collagen synthesis in human dermal fibroblasts [74].

**Figure 4:**
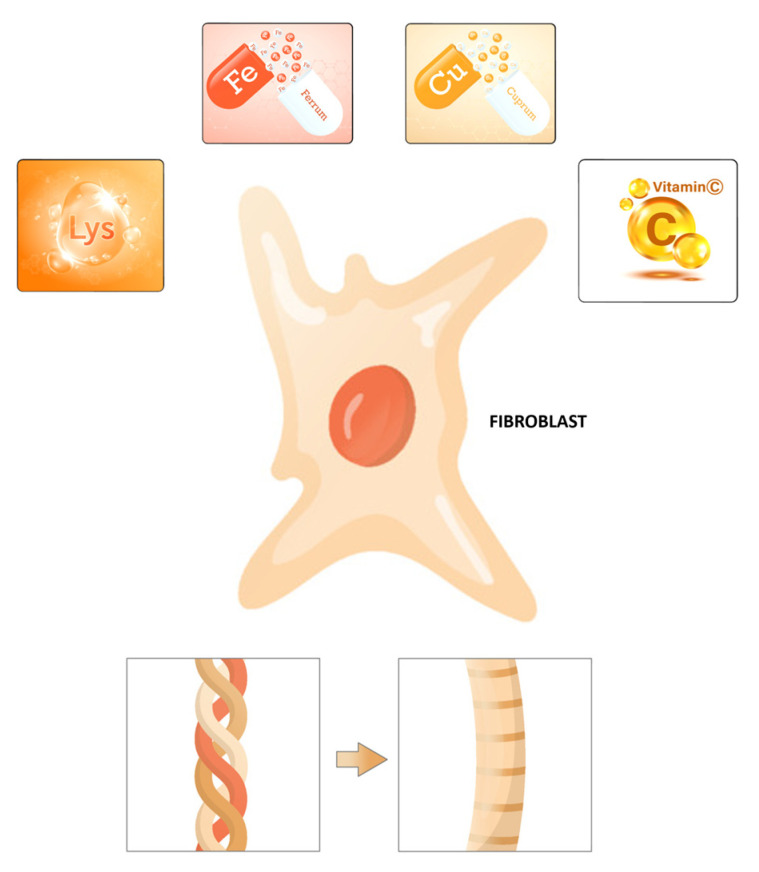
**Micro- and macronutrients for collagen biosynthesis:** Collagen production requires both micronutrients and macronutrients. Collagen fibers are made from fibroblasts, which are shown schematically here. Lysine, a macronutrient, is highlighted with vitamin C, copper, and iron, all of which are micronutrients.

Previously, the content of essential amino acids (isoleucine, leucine, lysine, methionine, phenylalanine, threonine, valine) and some mineral elements, including potassium, calcium, magnesium, iron, copper, zinc, and phosphorus, has been measured in dry beans [75]. For example, it was reported that lysine and Fe (68.9 to 152 mg per kg) are the most abundant nutrients in a Chilean bean sample [75]. In addition, an amino acid content analysis of native bean populations showed high content of lysine, arginine, and histidine (basic amino acids). Proline was the predominant non-essential amino acid found in kidney bean cultivars [76]. In contrast, glycine is detected in low concentrations in beans [10]. The human body can create hydroxylysine from a dietary amino acid, lysine, an essential amino acid nutrient that is indispensable to humans and animals and present in most protein food sources (Figure 4) [77]. Lysine content is especially high in fish, meats, and dairy products and higher than most other amino acids in wheat germ, legumes, nuts, quinoa, peas, and beans [78,79]. The composition and functional properties of proteins of common dry beans have been studied in terms of amino acid composition [80]. Aspartic and glutamic acids were predominant, and significant quantities of essential amino acids were detected, particularly leucine, lysine, and phenylalanine. Chemically, the characterization of amino acids from a saline and soluble protein concentrate, prepared from four common dry beans, has adequate amino acid contents with reactive side chains, which are part of different covalent cross-links in collagens: (1) aspartic and glutamic acids (negatively charged); (2) lysine and arginine (positively charged); (3) threonine, tyrosine, and cysteine (polar/hydrophilic); and (4) leucine, isoleucine, valine, and alanine (nonpolar/hydrophobic).

Lysine content is especially high in common beans (Table 2) and the lysine percentage in the protein collagen constitutes 3 to 4% of the total amino acid composition. However, it plays a crucial role in the formation of cross-links between collagen molecules, which are essential for the construction of collagen fibrils and fibers. In order to facilitate the functioning of this particular biological process, it is necessary for a subset of lysine molecules to undergo hydroxylation, while another subset undergoes oxidation, resulting in the formation of aldehyde compounds [81].

## 5. Cofactors for Collagen Biosynthesis

If the organism has all amino acid building blocks (Gly, Lys, Pro), but the number of cofactors is low (vitamin C, iron, and copper), the enzymes to form the protein collagen in the body may not function appropriately (PH, LH, and LOX). Micronutrients, including vitamins and minerals, regulate several biological events and are considered essential cofactors for collagen biosynthesis in humans [21]. Vitamin C and lysine, an essential amino acid, are key micro- and macronutrients that synergistically provide healthy collagen production (Figure 4) [86]. In addition, vitamin C has a vital role in wound healing by increasing procollagen and collagen [20,87]. For example, skin fibroblasts depend on vitamin C, an essential cofactor for synthesizing collagen [86]. Vitamin C is a water-soluble, effective antioxidant that has been shown to play an additional role in wound healing by improving procollagen production [88,89]. In addition, vitamin C supplementation in animals resulted in improved collagen synthesis in vivo. Besides stabilizing the collagen molecule with hydroxylation, vitamin C also stimulates collagen mRNA production and protein synthesis with fibroblasts [90]. Vitamin C is a cofactor for proline and lysyl hydroxylases that stabilizes the collagen molecule’s tertiary structure and promotes collagen gene expression. The dependence of the collagen hydroxylase enzymes on vitamin C has been demonstrated in several studies with fibroblast cells in vitro, with both decreased total synthesis and decreased cross-linking when vitamin C is absent [86,91]. Phytic acid, tannins, ascorbic acids, thiamin, and some minerals including K, Ca, Mg, Zn, Fe, and Cu have been detected in the common bean [8,15,80]. Copper has several functions; it is critical for iron absorption because it is part of ceruloplasmin, a protein that oxidizes Fe^2+^ to Fe^3+.^ In addition, copper is a recognized cofactor of lysyl oxidase enzymes, which are critical for collagen maturation [67]; furthermore, it is required to reduce Fe^3+^ back to Fe^2+^ to maintain the functioning of proline and lysyl hydroxylase, which is critical for enhancing tissue thickness (Figure 2). Therefore, both copper and iron are essential components in this process.

Polyphenols are secondary metabolites found in plants, fruits, vegetables, floral tissues, stems, bark, and roots and are widely known for their antioxidative capacity [92]. Flavonoids are the primary source of phenolic compounds and are associated with important health benefits [93]. The primary polyphenols found in beans are flavonoids, and their chemical nature has been extensively studied [94]. The content of total polyphenols and flavonoids has been quantified in beans and it ranges between 0.1 to 3.8 mg (mg GAE/g DW) and 0.2 to 7.0 mg (mg RE/g DW). Moreover, flavonoids can stimulate fibrillar collagen production in a mouse fibroblast model [95]. For example, anthocyanidins and catechin, natural plant pigments found in fruits, flowers, and some vegetables, have been demonstrated to stabilize collagens [25,96,97,98]. Also, the effect of an aqueous extract of *Phaseolus vulgaris* has been related to a positive influence on the properties of tendon collagen in streptozotocin-diabetic rats [5]. Therefore, using beans’ bioactive compounds as cofactors for enzymatic collagen cross-linking could promote the stabilization of collagen molecules. However, additional studies are necessary to clarify the effect of *Phaseolus vulgaris* extracts on the enzymatic collagen cross-linking.

## 6. Collagen Sources and Production

Collagen, which is the predominant protein within the human body, plays a crucial role in conferring structural integrity and providing support to a diverse array of tissues, encompassing the skin, bones, tendons, and ligaments [61]. The human body has an endogenous capacity to synthesize collagen, but it can also be acquired exogenously, predominantly through dietary means [99]. The primary origins of collagen protein are derived from animal tissues, particularly the skin. Collagen is found in significant quantities inside the dermal layers of various animal species, with fish and poultry being particularly rich sources [100]. The skin of fish, specifically, possesses a high concentration of type I collagen, which has advantageous effects on the health of human skin. It has been shown that the extraction of collagen from bones and bone marrow can be achieved by means of a boiling procedure, thus rendering bone broth a widely favored reservoir of collagen [101]. Moreover, connective tissues, namely ligaments and tendons, which are frequently disregarded during food preparation, possess a high abundance of collagen, rendering them highly valuable as sources of this protein. Certain fish scales, such as those of tilapia, contain a significant amount of collagen [102]. These scales are sometimes processed into collagen supplements. Fish collagen is known for its smaller particle size, making it easier to digest and absorb [103].

Collagen is a protein that is predominantly found in animal tissues, making it challenging to obtain directly from vegetarian sources. However, there are some alternatives and plant-based options that may contribute to collagen production or provide similar benefits for people who do not use animal products (Figure 4) [104]. Although plants do not naturally contain collagen, specific food items can stimulate the body’s endogenous collagen synthesis, which are denominated Collagen-Boosting Foods [105]. In this type of products, collagen synthesis is promoted by foods rich in collagen cofactors, which are essential micronutrients found in abundance in grape seeds and tomato extracts [106]. These products promote collagen synthesis in the body or offer other health benefits. For example, plant-based foods rich in amino acids like glycine, proline, and hydroxyproline, such as legumes, beans, lentils, tofu, tempeh, quinoa, nuts, and seeds, support the body’s own collagen production [107]. Vitamin C-rich foods that are essential for collagen synthesis, including fruits and vegetables like citrus fruits, strawberries, and kiwi, among others, can help support collagen formation. Antioxidants protect collagen from damage caused by free radicals; thus, foods like berries rich in antioxidants can help maintain collagen production. Additionally, collagen and cofactors are encapsulated for ease of consumption, enabling individuals to take advantage of its benefits without making substantial dietary adjustments [108].

In conclusion, the collagen protein and cofactors come from animal tissues, fish scales and bones, cartilage, plant-based diets, and marine sources; however, it is essential to understand that while these alternatives can help collagen synthesis, they may not provide identical biological effects to collagen obtained directly from animal sources. New collagen methods for extraction may emerge as research advances, giving people more alternatives to boost their collagen consumption and improve their health. As research and technology advance, more innovative solutions for producing collagen-like products from vegan sources will be developed in the future, especially considering the growing interest in plant- and collagen-derived protein as supplemental or replacement dietary sources because of widespread environmental sustainability concerns [109,110].

## 7. Collagen Precursor Bioavailability

The term “bioavailability” describes how well a nutrient is absorbed and used by the body after ingestion. The collagen synthesis is facilitated with foods rich in proline, glycine, and lysine, which are amino acids found in abundance in common beans. Proteins, peptides, minerals (e.g., copper and iron), vitamins (e.g., vitamin C), and flavonoids (catechin) that are essential for the synthesis, secretion, and cross-linking of collagen are all found in beans. Protein-building micro- and macronutrients found in abundance in plants, including beans, are suitable for vegan diets in which the consumption of animal-derived foods is restricted or nonexistent [111]. Bean proteins are typically thought to have good bioavailability. However, the bioavailability of proteins can be influenced for a number of variables, including cooking methods, anti-nutrients (e.g., phytates), and individual differences in digestion and metabolism [112]. Glycine-rich peptides, for example, are short chains of amino acids containing a significant amount of glycine. Glycine is an essential amino acid, and its bioavailability is generally high when obtained from protein-rich sources [60].

Beans are a good source of minerals, including iron and copper. However, plant-based sources of iron (non-heme iron) are less readily absorbed compared to heme iron found in animal products. To enhance iron bioavailability from beans, it is recommended to integrate foods abundant in vitamin C into the dietary regimen, such as citrus fruits like orange that help improve non-heme iron absorption. Copper, on the other hand, is generally well-absorbed from plant sources, and the bioavailability is not a major concern in vegan diets, especially if the diet is balanced and varied [9].

Finally, catechin is a type of flavonoid present in cocoa beans and some types of green beans. However, these phenolic compounds could be degraded during processing of beans. The bioavailability of catechins can be influenced by various factors, including the food matrix, gut microbiota, and interactions with other dietary components. Still, catechins from beans are generally considered to have reasonable bioavailability and a recent study proved that the absorption efficiency of the cocoa phenolic compounds was between 87.9 and 97.4%, while in the coffee compounds, it was 100%. Thus, the high bioavailability and a valuable antioxidant capacity of these beans were confirmed [113,114].

## 8. Protein and Peptide Digestion and Absorption

**Figure 5:**
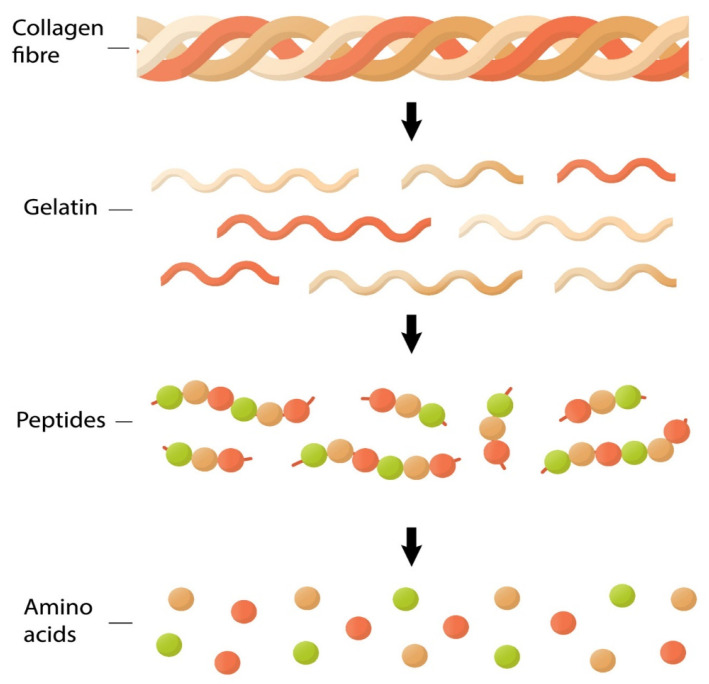
**Chemical composition of different types of animal collagen that exist in the actual market.** Collagen fiber is formed with the basic unit (named triple helix). Gelatins correspond to denatured collagen using boiling. Collagen peptides are short chains of amino acids obtained from native collagen. Also, the figure represents amino acids glycine (red circle), lysine, hydroxylysine, proline (green circle), and hydroxyproline (orange circle) that are components of the primary structure of collagen molecules.

Proteins and peptides, including glycine-rich peptides, are large molecules made up of amino acids (Figure 5). The process of protein digestion and absorption in the gut involves several steps. Once the proteins are in the stomach and exposed to acidic conditions, the enzymes break down the protein molecules into smaller peptides. Enzymes like pepsin cleaves proteins at particular aminoacidic sites, producing fragments of peptides. Other enzymes including trypsin, chymotrypsin, and carboxypeptidases further break down the peptide fragments into even smaller peptides and individual amino acids in the small intestine. Peptides, including glycine-rich peptides, are broken down into their individual amino acids by these peptidases. The final step is the absorption of amino acids and small peptides across the lining of the small intestine into the bloodstream [115]. Amino acids are absorbed via active transport mechanisms, while small peptides are absorbed through specialized peptide transporters, mainly by the SLC15A gene subfamily (peptide transporters, PEPTs). Once absorbed, amino acids and peptides are transported through the bloodstream to various tissues and organs in the body, where they are utilized for protein synthesis like collagen, energy production, and other physiological functions [116].

Oligopeptides are absorbed through specialized peptide transporters placed on the surface of the small intestine cells. These transporters are part of the SLC15 family of transport proteins, particularly peptide transporters PEPT1 and PEPT2. The role of PEPT1 in the small intestine is to facilitate the uptake of dipeptides and tripeptides derived from the digestion of dietary proteins, and PEPT2 in the kidney is involved in the reabsorption and conservation of filtered peptides from the urine back into the bloodstream, preventing excessive peptide loss [117,118].

The effects of collagen derived directly from animal sources are different from vegan sources primarily due to the structural differences and amino acid composition between animal-based collagen and plant-based alternatives. The amino acid composition of collagen from animal sources contains specific amino acids like glycine, proline, and hydroxyproline in the exact ratios needed to form the characteristic triple-helix structure of collagen. These amino acids are essential for providing the unique properties of collagen, such as its strength and stability. On the other hand, plant-based sources may lack some of these specific amino acids or may not have them in the same optimal proportions, making it challenging to replicate the exact properties of animal-derived collagen [109]. Other differences are that animal-based collagen typically contains types I, II, and III collagen, which are the most abundant types in the human body, while plant-based sources generally do not contain these specific collagen types. Regarding the absorption and bioavailability, the animal-based collagen is usually more easily absorbed and has higher bioavailability than plant-based alternatives. This is because animal-derived collagen is very similar to human collagen, making it easier for the body to use it. If the manufacturing and processing is considered, collagen derived from animal sources goes through specific extraction and processing methods to maintain its structure and functionality [119]. Recreating this complex protein structure from plant-based sources can be challenging and may result in products with different characteristics and properties. Moreover, collagen from animal sources often comes with other essential nutrients like minerals and vitamins that support overall collagen synthesis and tissue health. These nutrients may not be present or may be present in different quantities in plant-based collagen alternatives. More information can be found in the recent review where the sources and potential cosmetic applications of collagen are discussed [27].

In the context of Hypertension, many legumes, such as the common bean, have been found to contain bioactive peptides that exhibit inhibitory effects against angiotensin-converting enzyme (ACE) activity [120]. These peptides can be recovered using different methods, including solvent extraction, enzymatic hydrolysis, or fermentation. The polypeptides in question include amino acids, including glycine and proline, which have the potential to enhance collagen synthesis. Nevertheless, further investigations are required to validate this notion.

## 9. Conclusions

While plant-based collagen alternatives can provide several benefits, they are not true collagen replacements and may not have the same effects as animal-derived collagen. However, a balanced diet rich in various nutrients and amino acids can still support healthy skin, joint function, and overall tissue health, and contribute to the synthesis of collagen for the human body. The consumption of common beans can help maintain collagen production because they contain amino acids and cofactors. Common beans contain high levels of lysine, an essential amino acid deficient in most cereals [13]. Lysine is especially important because it is essential for building cross-links between the molecules to construct the fibrils and fibers of collagens (necessary for healthy tissue growth). Amino acids are key components of human and animal nutrition, as part of a protein-containing diet and as supplemented with individual products [121]. Amino acids play a crucial role in medical nutrition, particularly in parenteral nutrition with high purity requirements for infusion-grade products. Thus, the ability to isolate L-Lysine from the common bean presents a chance for animal feed and human supplementation to develop functional collagen for body building for vegetarians who choose to follow a plant-based diet. In addition, like other food, legumes like common beans contain numerous bioactive compounds, such as phenolic-rich substances that play crucial metabolic functions in humans and animals. Additional studies are necessary to improve our understanding of earlier undefined roles of micro- and macronutrients of *Phaseolus vulgaris* extracts for their beneficial effects on collagen biosynthesis and enzymatic cross-linking. Beans have an important role in the protein food group due to their high protein content. For both vegetarian and vegan diets, beans play a key role. The health perks of beans, or more broadly, legumes, are many. These include better heart health, improved digestion, and help with weight management. They are rich in plant-based protein and one of the easiest ways to incorporate plant protein into a vegetarian diet [122]. With the excellent nutritional profile from beans, particularly their high quality of amino acids with lysine and leucine, they are highlighted as an excellent source of vegetable protein, which even enhances the collagenous synthesis, renewing cartilage, bone tissue, and skin cells, and even repairing damage structures. Bean content includes enough quantity of fiber and antioxidants per 100 g; therefore, this characteristic is also important to potential health in vegan people. In addition, this feature is also important for the potential health of vegan people. On the other hand, the contribution of micronutrients from beans, including iron, potassium, phosphorus, calcium, and vitamins of the B, E, and K complex, helps prevent nutritional deficiencies in vegetarian and vegan diets. In summary, beans can be an excellent alternative protein source for vegans, and they provide various other essential nutrients with acceptable bioavailability. However, to maximize nutrient absorption, it is essential to consume a diverse and well-balanced diet, including other plant-based foods rich in vitamins, minerals, and antioxidants, alongside beans.

There are some limitations regarding collagen synthesis. While it is known that certain amino acids and cofactors are required for collagen formation, the exact mechanisms and interactions involved in collagen synthesis are still not fully understood. Further research is needed to uncover the complexities of this process. Another concern is the variability in bean nutrient content. Common beans can vary in nutrient content based on factors such as variety, growing conditions, and processing. This variability can make it challenging to establish consistent nutritional guidelines for health professionals or consumers. Moreover, human bodies vary in their ability to absorb and utilize nutrients, which can be influenced by genetic factors, individual health conditions, or dietary choices. The effectiveness of beans as a source of amino acids and cofactors for collagen synthesis may differ from person to person. Thus, more studies on nutrigenomics should be performed [120].

The concept of using foods like common beans not only as a source of nutrients but also as functional foods or nutraceuticals with specific health benefits is a growing trend [123]. Researchers may explore the bioactive compounds in beans that have a direct impact on collagen and overall health. With a deeper understanding of collagen synthesis and the role of dietary components, future trends may lead to the development of nutritional therapies to support conditions related to collagen, such as skin health, wound healing, and joint health.

## Figures and Tables

**Table 2:** Content of amino acids and inorganic cofactors in *Phaseolus vulgaris* L. related to the collagen biosynthesis.

Amino Acids and Cofactors	Average Amount	References
Glycine	0.97 g/100 g	[82,83]
Proline	0.87 g/100 g	[82,83]
Lysine	1.0–2.2 g/100 g	[84,85]
Iron	6.52–10.4 mg/100 g	[16]
Copper	0.93–1.21 mg/100 g	[16]

## Data Availability

Not aplicable.
